# 710. Comparison of Initial CXR to CT in Patients (pts) with Hematologic Malignancy (HEM) and Documented Symptomatic Pulmonary Mucormycosis (PM)

**DOI:** 10.1093/ofid/ofab466.907

**Published:** 2021-12-04

**Authors:** Alexander Franklin, Dierdre B Axell-House, Amy Spallone, Jeffery Tarrand, Dimitrios P Kontoyiannis

**Affiliations:** 1 MD Anderson, Houston, Texas; 2 Baylor College of Medicine, Houston, TX; 3 The University of Texas MD Anderson Cancer Center, Houston, TX

## Abstract

**Background:**

There is a spectrum of pulmonary disease burden in pts with HEM and PM. There have not been any data comparing the sensitivity and findings of initial Chest X-Ray (CXR) and chest CT in these pts.

**Methods:**

We compared the findings of the initial CXR and CT in all pts with proven or probable PM via EORTC/MSG criteria. We included only pts who had pulmonary symptoms and who had both CXR and CT within 5 days of each other and within seven days of symptom onset or date of culture at MD Anderson Cancer Center from April 2000 and April 2020. We collected data regarding demographics, status of HEM, clinical presentation, frequency and findings of BAL and imaging findings, mold-active prophylaxis and treatment regimens, and mortality. CXR findings were classified as normal or abnormal, and if abnormal sub-classified as mass-like/consolidative, nodular, cavitary or heterogenous/non-specific. CT findings were classified in a similar manner.

**Results:**

We Identified such 39 pts with PM who had both CXR and CT within 5 d. All pts had positive CT. Five pts (13%) had a negative CXR. The majority of pts 28 (72%) were neutropenic (neutrophil count < 500). The most common CXR findings were consolidation or mass-like lesions (56%), followed by patchy, heterogenous or non-specific findings (33%) and nodules (13%). Only 3% had cavitary lesions. Similarly, consolidation or mass-like lesions were the most common finding on CT (69%), followed by nodular lesions with or without ground glass halos (56%). Cavitary lesions and/or reverse halo sign (RHS) were common (31%) on CT. Patients with normal CXR vs those with abnormal CXR were comparable in all clinical parameters we collected. The median survival from time of symptoms onset for all pts was 45 days. There was a trend for lower 42 day mortality in pts with normal CXR (20% vs 47%, P=.253).

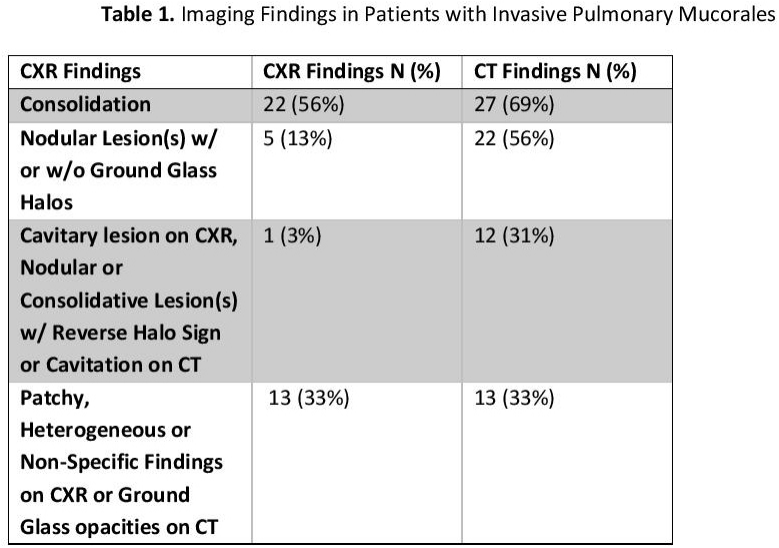

**Conclusion:**

A negative CXR does not preclude PM, especially in neutropenic pts. A CT is recommended for better sensitivity and although there was concordance in CXR with CT findings in some chest abnormalities (mass, consolidation), CT more commonly revealed nodules and signs highly suggestive of PM such as RHS. Although small numbers precluded a robust comparison, it is possible that HEM pts with PM and negative initial CXR have better prognosis, perhaps reflecting a lower burden of pulmonary involvement

**Disclosures:**

**Dimitrios P. Kontoyiannis, MD**, **Astellas** (Consultant)**Cidara Therapeutics** (Advisor or Review Panel member)**Gilead Sciences** (Consultant, Grant/Research Support, Other Financial or Material Support, Honoraria)

